# High-Precision Time-of-Arrival Estimation in HF Sensor Networks via Multipath Separation and Independent Tracking

**DOI:** 10.3390/s26051640

**Published:** 2026-03-05

**Authors:** Qiwei Ji, Huabing Wu

**Affiliations:** 1 National Time Service Center, Chinese Academy of Sciences, Xi’an 710600, China; jiqiwei21@mails.ucas.ac.cn; 2 University of Chinese Academy of Sciences, Beijing 100049, China; 3 Key Laboratory of Time Reference and Applications, Chinese Academy of Sciences, Xi’an 710600, China

**Keywords:** time-of-arrival (TOA) estimation, multipath separation, ionospheric propagation, HF sensor networks

## Abstract

High-frequency (HF) sensor networks play an irreplaceable role in remote sensing and emergency communications but suffer severely from ionospheric multipath interference, which degrades Time-of-Arrival (TOA) estimation accuracy. Conventional methods, such as the Generalized Cross-Correlation (GCC) and standard Delay-Locked Loops (DLL), often treat multipath components as noise, leading to significant measurement bias in dynamic environments. To address this, we propose a Multipath Separation and Independent Tracking (MSIT) architecture. This framework transforms multipath interference into valuable observables by establishing a closed-loop synergy: a Maximum Likelihood Estimation (MLE)-based module iteratively separates signal components, while parallel tracking loops update phase and delay parameters. Additionally, a super-resolution MUSIC algorithm is employed for initialization to resolve sub-chip multipath components. Simulations demonstrate that under disturbed channel conditions, the MSIT method achieves a mean delay estimation error reduction of about two orders of magnitude relative to the GCC method. Furthermore, field experiments on the Xi’an–Ürümqi link demonstrate its capability to stably resolve and track multiple propagation paths in real-world environments. This approach significantly enhances the measurement precision and reliability of HF sensing systems.

## 1. Introduction

High-frequency (HF) skywave communication utilizes ionospheric reflection to achieve over-the-horizon (OTH) transmission [1,2]. Due to this unique propagation mechanism, HF systems play an indispensable role in emergency response, military command, and remote sensing networks [3,4,5]. In these applications, precise Time-of-Arrival (TOA) estimation is a fundamental requisite for accurate source localization and channel characterization. However, the ionosphere functions as a dispersive, time-varying medium in which multipath propagation generates multiple signal replicas with closely spaced differential delays [1,3,6]. Recent studies have extensively characterized these complex channel conditions—ranging from high-dynamic hypersonic vehicle links [3] to massive MIMO-OFDM systems [7]—underscoring the persistent challenge of channel estimation in dense multipath environments. From a signal processing perspective, this phenomenon introduces significant ambiguity; traditional methods typically treat secondary multipath components as self-interference, which distorts the correlation function and biases the primary TOA measurement [1,3,4].

Conventional signal processing techniques often fail to maintain measurement integrity in such dynamic environments [2,5,8,9]. The Generalized Cross-Correlation (GCC) method, for instance, predicates its performance on a quasi-static, single-path channel model, resulting in substantial estimation errors during periods of rapid ionospheric fluctuation or mode hopping [9,10,11]. Similarly, traditional Delay-Locked Loops (DLLs) are fundamentally constrained by the Rayleigh resolution limit [2,12]. Because DLLs track the composite envelope of overlapping signals, they are unable to resolve intra-chip multipath components—delays shorter than one chip duration—which leads to persistent tracking bias. Furthermore, although subspace algorithms such as MUSIC offer super-resolution capabilities, their high computational complexity and sensitivity to noise render them impractical for continuous, real-time tracking in low-SNR conditions [13,14,15].

Expanding upon our prior research on adaptive modulation tracking [2], this paper introduces the Multipath Separation and Independent Tracking (MSIT) architecture. In contrast to conventional approaches that prioritize multipath suppression, MSIT treats each propagation path as a distinct and valuable observable. The framework establishes a closed-loop synergy: a Maximum Likelihood Estimation (MLE)-based separation module iteratively decomposes the raw data into “clean” streams, which are subsequently processed by parallel tracking loops. These loops provide real-time updates for phase and delay parameters, thereby dynamically refining the separation process. To ensure high-precision tracking, a MUSIC-based initialization strategy is employed at the acquisition stage to resolve sub-chip multipath components, effectively transforming complex interference into a set of resolvable signal sources.

## 2. Signal Model

To accurately evaluate the proposed delay estimation framework, we establish a signal model based on the ITU-R Watterson channel, which is the standard for simulating narrowband HF ionospheric propagation [16]. The channel is modeled as a tapped-delay-line where the received signal $y\left(t\right)$ is a superposition of N  multipath components and additive white Gaussian noise (AWGN) n(t):

(1) $$y\left(t\right)=\;\sum_{i=1}^{N}G_{i}\left(t\right)s\left(t-\tau_{i}\right)+n\left(t\right),$$

where $\tau_{i}$ denotes the propagation delay of the i-th path. $G_{i}(t)$ is the channel gain function of the *i*-th path, which describes the effects of small-scale fading characteristics on shortwave signals during transmission, such as Doppler spread, multipath fading, and Doppler frequency shift. $G_{i}(t)$ is defined in a complex form with mutually orthogonal real and imaginary parts:

(2) $$G_{i}\left(t\right)=c_{i}\left(t\right)e^{j\varphi_{i}}=g_{i,1}\left(t\right)+jg_{i,2}\left(t\right),$$

where $c_{i}\left(t\right)$ is the amplitude of the path, $\varphi_{i}$ is the phase; $g_{i,1}\left(t\right)$ and $g_{i,2}\left(t\right)$ are two independent and identically distributed (i.i.d.) real Gaussian random processes. According to the Central Limit Theorem, they are respectively expressed as:

(3) $$g_{i,1}\left(t\right)=\underset{Q_{1}\to \infty }{lim}\sum_{n=1}^{Q_{1}}c_{1,n}\;cos\varphi_{1,i}=\underset{Q_{1}\to \infty }{lim}\sum_{n=1}^{Q_{1}}c_{1,n}\;cos\left(2\pi f_{d1,n}t+\theta_{1,n}\right),$$

(4) $$g_{i,2}\left(t\right)=\underset{Q_{2}\to \infty }{lim}\sum_{n=1}^{Q_{2}}c_{2,n}\;cos\varphi_{2,i}=\underset{Q_{2}\to \infty }{lim}\sum_{n=1}^{Q_{2}}c_{2,n}\;cos\left(2\pi f_{d2,n}t+\theta_{2,n}\right),$$

in the above expressions, $i\;=\;1,2,\ldots ,Q_{1}$ or $Q_{2}$; $Q_{1}$ and $Q_{2}$ represent the number of sinusoidal superpositions for the two real random processes, respectively; $c_{1,n}$ and $c_{2,n}$ denote random path fading; $\varphi_{1,i}$ and $\varphi_{2,i}$ are random phases; $f_{d1,n}$ and $f_{d2,n}$ represent Doppler frequency shifts; $\theta_{1,n}$ and $\theta_{2,n}$ are random phase shifts uniformly distributed over [0,2).

The transmitted signal $s\left(t\right)\;$ employs 8-Phase Shift Keying (8PSK) modulation, selected for its spectral efficiency in HF bands. The chip rate of the signal is set to 2.4 kHz, corresponding to a chip duration of Tc=1/2.4 kHz≈0.417 ms. For the purpose of the tracking algorithm design, the channel gain $G_{i}\left(t\right)\;$ for a single magneto-ionic component can be decomposed into an amplitude term and a Doppler rotation term. Consequently, the received signal model is reformulated to explicitly reveal the parameters to be tracked (delay, frequency, and phase):

(5) $$y(t)=\sum_{i=1}^{N}\alpha_{i}p\left(t-\tau_{i}\right)cos(2\pi (f_{c}+f_{d,i})t+\theta_{i})+n\left(t\right),$$

In this expression, $\alpha_{i}$ represents the time-varying amplitude of the i-th path. $p\left(t\right)$ is the baseband shaping function of the 8PSK symbols. $f_{c}$ is the carrier frequency, and $f_{d,i}$ is the Doppler frequency shift associated with the i-th path. $\theta_{i}$ denotes the carrier phase.

## 3. Principle of MSIT

### 3.1. MSIT Parameter Initialization

Accurate initialization of multipath parameters is essential for subsequent signal tracking. Conventional correlation-based methods, however, are often unable to resolve intra-chip multipath components due to the inherent Rayleigh resolution limit. To overcome this limitation, we employ the Multiple Signal Classification (MUSIC) algorithm [13,14,15], which transforms time-domain delay estimation into frequency-domain spectral analysis. This approach achieves super-resolution, enabling the precise identification of signal paths with sub-chip spacing.

The initialization process comprises two stages: coarse frequency acquisition and fine delay estimation. First, the analytic signal of the received signal y(t) is obtained via the Hilbert transform to eliminate phase ambiguity. Coarse frequency compensation is then implemented via FFT-based correlation, where the received signal is multiplied by local reference carriers at steps $f_{search}$ and correlated with the transmitted code in the frequency domain, as shown in Formula (6):

(6) $$Z(k,f_{search})=IFFT(FFT(y(t)\cdot e^{-j2\pi f_{search}t})*FFT(p_{loc}(t))),$$

The frequency $f_{d\_ac}\;$ corresponding to the maximum correlation peak is selected as the coarse carrier frequency offset. The received signal is then demodulated by $f_{d\_ac}\;$ to obtain the baseband signal $y_{bb}(t)$, which serves as the input for the MUSIC algorithm.

The core of the proposed initialization method is to construct “frequency domain snapshots” to apply subspace methods. According to the convolution theorem, the frequency spectrum $Y\left(f\right)\;$ of the baseband signal is the product of the transmitted signal spectrum X(f) and the channel transfer function H(f). Thus, the discrete channel response at the k-th frequency bin can be estimated by:

(7) H(k)=Y(k)∗X(k), k∈Ω,

where Ω represents the set of frequency indices within the effective signal bandwidth (main lobe). The vector $h=[H(1),H(2),\ldots ,H(M){]}^{T}$ constitutes the frequency-domain observation vector, where M is the number of valid frequency points. In this model, different frequency components are analogous to array elements in Direction of Arrival (DOA) estimation, and the propagation delay τ corresponds to the direction of arrival.

Since multipath signals in HF channels are fully coherent (originating from the same source), the covariance matrix of h is rank-deficient, which causes standard MUSIC to fail. To decorrelate the signals, we employ the Forward-Backward Spatial Smoothing (FB-SS) technique. We define a sub-array length L and divide the vector h into overlapping sub-vectors. The forward covariance matrix $R_{f}$ and backward covariance matrix $R_{b}\;$ are computed as:

(8) $$R_{f}=1/P\sum_{i=1}^{P}h_{i}h_{i}^{H},R_{b}=1/P\sum_{i=1}^{P}(Jh_{i}^{*}){\left(Jh_{i}^{*}\right)}^{H},$$

where P is the number of smoothing steps, $h_{i}$ is the i-th sub-vector, and J is the exchange matrix (a matrix with ones on the anti-diagonal and zeros elsewhere). The final smoothed covariance matrix is $R=(R_{f}+R_{b})/2$.

By performing Eigenvalue Decomposition (EVD) on R, we obtain the signal subspace $U_{s}$ and the noise subspace $U_{n}$. The number of multipath components N  is estimated based on the ratio of adjacent eigenvalues. It is acknowledged that information-theoretic criteria, such as the Akaike Information Criterion (AIC) or Minimum Description Length (MDL) [17,18], offer theoretically robust source enumeration, especially in low-SNR regimes. However, these methods involve computationally intensive logarithmic and trace operations. Considering the operational SNR range of HF sensor networks and the requirement for hardware efficiency on FPGA platforms, the Eigenvalue Ratio method provides a satisfactory trade-off between accuracy and computational simplicity. The MUSIC pseudospectrum function $P_{MUSIC}\left(\tau \right)$ is then constructed by projecting the delay steering vector $a\left(\tau \right)$ onto the noise subspace:

(9) $$P_{MUSIC}(\tau )=1/a^{H}(\tau )U_{n}U_{n}^{H}a(\tau ),$$

where the steering vector is defined as $a(\tau )=[e^{-j2\pi f_{1}\tau },e^{-j2\pi f_{2}\tau },\ldots ,e^{-j2\pi f_{L}\tau }{]}^{T}$, and $f_{1},\ldots ,f_{L}$ correspond to the physical frequencies of the subarray.

By searching for the peaks of $P_{MUSIC}(\tau )$ in the delay domain, the delays of all multipath components ${\hat{\tau }}_{1},{\hat{\tau }}_{2},\ldots ,{\hat{\tau }}_{N}$ are accurately determined, providing precise initial values for subsequent tracking.

### 3.2. Multipath Separation and Independent Tracking Principle

**Figure 1:**
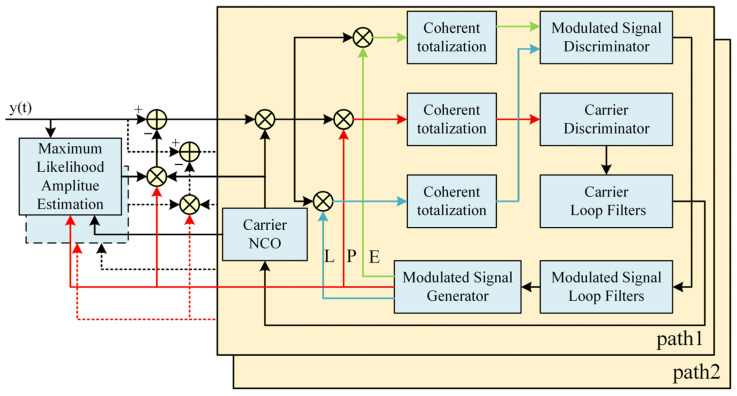
Schematic Diagram of Multipath Tracking Principle.

To overcome the limitations of traditional single-path tracking in resolving closely spaced multipath components, we propose a Multipath Separation and Independent Tracking (MSIT) architecture. In contrast to conventional approaches that treat multipath components merely as interference, the MSIT framework regards each path as a distinct signal of interest [19,20]. As illustrated in Figure 1, the system operates through a synergistic closed-loop mechanism: tracking loops provide real-time parameter estimates to the separation module, which subsequently employs Maximum Likelihood Estimation (MLE) to reconstruct interference-free signals for each loop, thereby iteratively enhancing estimation precision [21].

#### 3.2.1. Signal Reconstruction and Interference Cancellation

The core innovation of the MSIT architecture lies in its iterative interference cancellation mechanism. The received multipath signal is modeled as a linear superposition of N  path components:

(10) $$r\left(t\right)=\sum_{i=1}^{N}\alpha_{i}\cdot s\left(\tau_{i},\theta_{i}\right)+n\left(t\right),$$

where $\alpha_{i}$ is the complex amplitude (containing both channel gain and residual phase error); $n\left(t\right)$ is Gaussian white noise. The basis vector $s\left(\tau_{i},\theta_{i}\right)$ represents the normalized reference signal for the i-th path, constructed using the delay $\tau_{i}\;$ from the Delay-Locked Loop (DLL) and the carrier phase $\theta_{i}\;$ from the Phase-Locked Loop (PLL):

(11) $$s\left(\tau_{i},\theta_{i}\right)=p\left(t-\tau_{i}\right)\cdot e^{j\left(2\pi f_{i}t+\theta_{i}\right)},$$

Here,$\;p\left(\cdot \right)\;$ is the 8PSK baseband symbol, and $f_{i}\;$ includes the carrier frequency and the Doppler shift tracked by the Frequency-Locked Loop (FLL).

To accurately reconstruct the signal, we must estimate the complex amplitude vector $\alpha ={\left[\alpha_{1},\;\alpha_{2},\;\ldots \;,\alpha_{N}\right]}^{T}$. Based on the Maximum Likelihood criterion, this is equivalent to minimizing the squared error between the received signal and the reconstructed model:

(12) $$\hat{\alpha }\;=\;arg\;min{\left\|r\;-\;H\alpha \right\|}^{2},$$

where $\left\|\cdot \right\|\;$ denotes the L2 norm of a vector; $H=[s_{1},\;s_{2},\;\ldots \;,s_{N}]$ is the design matrix composed of the reference signals for all paths. The optimal solution is given by the Least Squares (LS) estimator:

(13) $$\hat{\alpha }\;=\;{\left(H^{H}H\right)}^{-1}H^{H}r,$$

Once the complex amplitude ${\hat{\alpha }}_{i}\;$ is obtained, the signal component for the i-th path can be reconstructed as:

(14) $${\hat{w}}_{i}={\hat{\alpha }}_{i}\cdot s\left(\tau_{i},\theta_{i}\right),$$

This reconstructed signal ${\hat{w}}_{i}\;$ represents the best estimate of the i-th path’s contribution to the total signal. To achieve accurate tracking for a specific target path k, we employ Parallel Interference Cancellation (PIC). The interference term $I_{k}$ consists of the sum of all other reconstructed paths:

(15) $$I_{k}=\sum_{i=1,i\neq k}^{N}{\hat{w}}_{i},$$

The “clean” input signal $y_{k}\;$ for the k-th tracking loop is then obtained by subtracting this interference from the original received signal:

(16) $$y_{k}=r-I_{k}=r-\sum_{i=1,i\neq k}^{N}{\hat{w}}_{i},$$

This iterative interference cancellation and parameter update mechanism allow the algorithm to robustly track time-varying multipath signals. As the tracking loops converge, the basis functions $s\left(\tau ,\theta \right)\;$ become more accurate, leading to better amplitude estimation and cleaner interference cancellation, which in turn further improves tracking precision.

#### 3.2.2. Independent Single-Path Tracking and Delay Calculation

The separated “clean” signal $y_{k}\;$ serves as the input to the k-th independent tracking unit. This unit comprises a Carrier Tracking Loop (PLL/FLL) and a Code Tracking Loop (DLL), designed to maintain a continuous lock on the signal’s phase and delay.

The Carrier Tracking Loop removes the residual Doppler frequency offset and carrier phase error [20,22]. A two-quadrant arctangent discriminator is employed for the PLL, while a differential phase discriminator is used for the FLL [23]. A second-order digital loop filter adjusts the carrier Numerically Controlled Oscillator (NCO) to align the local carrier with the received signal, ensuring coherent demodulation.

The Code Tracking Loop (DLL) is responsible for the precise estimation of the Time of Arrival (TOA). It utilizes a normalized non-coherent “Early-minus-Late” envelope discriminator to detect the residual code phase error δτ [24,25]:

(17) $$D_{DLL}={(P}_{E}-P_{L})/{(P}_{E}+P_{L}),$$

where $P_{E}\;$ and $P_{L}\;$ represent the correlation power of the Early and Late branches, respectively. The discriminator output is filtered and used to drive the code NCO.

Unlike standard communication receivers that focus solely on synchronization, this study aims for high-precision physical delay estimation. The delay estimate is derived directly from the accumulated phase of the code NCO. In our implementation, the NCO tracks the absolute code phase (in units of chips). The physical propagation delay ${\hat{\tau }}_{k}\left[n\right]\;$ for the k-th path at time index n  is calculated by normalizing the accumulated code phase by the code sequence parameters:

(18) $${\hat{\tau }}_{k}\left[n\right]=\varphi_{code,k}\left[n\right]\cdot T_{seq}/N_{seq},$$

where $\varphi_{code,k}\left[n\right]$ is the cumulative code phase tracked by the NCO (in chips). $N_{seq}$ is the total number of chips in one transmitted code sequence. $T_{seq}$ is the duration of one complete code sequence.

## 4. Results

### 4.1. Simulation Results

To comprehensively evaluate the performance of the proposed shortwave multipath delay tracking algorithm within complex channel environments, simulation experiments were conducted utilizing the Watterson channel model. The transmitted signal consisted of 48 randomly generated integers within the interval ([0,8)) per transmission period, adopting the 8PSK modulation scheme with a symbol bandwidth of 2.4 kHz, a carrier frequency of 3.6 MHz, and a receiver sampling rate of 10.8 MHz. In the simulations, the reference delay is set to 7 ms, and each set of experimental data contains 10 signal periods.

#### 4.1.1. Performance Comparison Under Different Latitudes and Channel States

To comprehensively evaluate the adaptability of the proposed shortwave multipath delay tracking algorithm within complex channel environments, simulation experiments were conducted utilizing the ITU-R standard HF channel model constructed via MATLAB R2025b’s built-in stdchan function. The experiments covered three geographical scenarios (Low-latitude, Mid-latitude, and High-latitude) and three channel disturbance states (Quiet, Moderate, and Disturbed), totaling 9 typical shortwave propagation scenarios. The proposed Multipath Separation and Independent Tracking (MSIT) algorithm was compared with the traditional Single-path tracking algorithm (which tracks only the main peak delay without multipath separation) and the Generalized Cross-Correlation (GCC) algorithm.

To evaluate the estimator’s performance, the Cramér-Rao Lower Bound (CRLB) is included in the tables. Under the simulation conditions of 2.4 kHz bandwidth and 20 dB SNR, the theoretical CRLB is approximately 0.0023 ms.

1. Results in Low-Latitude Regions

**Figure 2:**
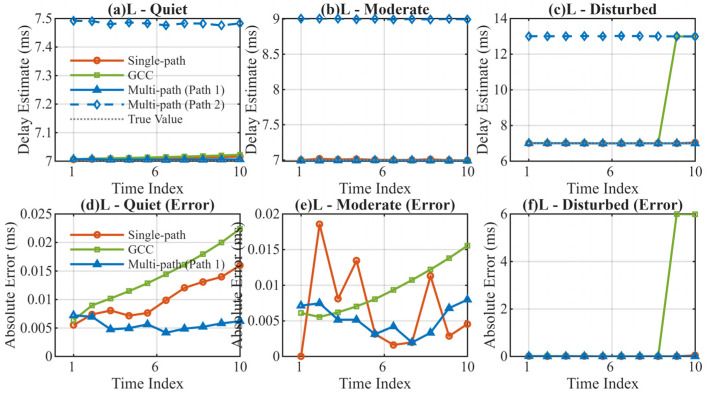
Delay Estimation Comparison in Low-Latitude Regions. (**a**) Delay estimation in low-latitude quiet channel; (**b**) Delay estimation in low-latitude moderate channel; (**c**) Delay estimation in low-latitude disturbed channel; (**d**) Absolute error in low-latitude quiet channel; (**e**) Absolute error in low-latitude moderate channel; (**f**) Absolute error in low-latitude disturbed channel.

The mean delay estimation errors for the three algorithms in low-latitude scenarios are presented in Table 1, with the corresponding delay tracking curves and error distributions shown in Figure 2.

The results indicate that while all three algorithms maintain relatively low errors under quiet and moderate conditions, the proposed MSIT algorithm achieves the highest precision. However, in the disturbed channel (LD), the error of the GCC algorithm rises sharply to 1.203 ms, whereas the MSIT algorithm maintains an extremely low error of 0.003 ms, demonstrating superior resistance to channel disturbances.

2. Results in Mid-Latitude Regions

**Figure 3:**
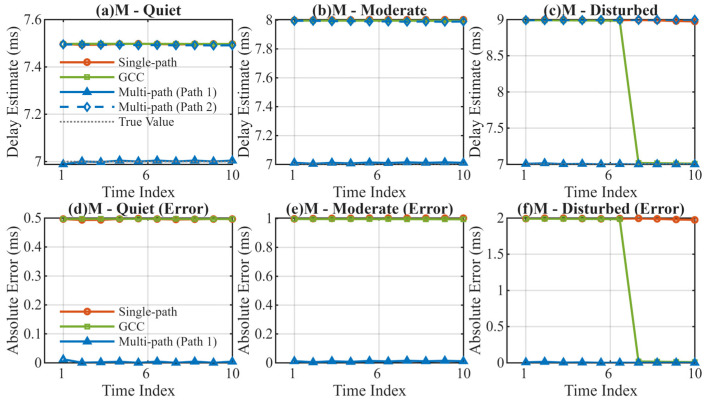
Delay Estimation Comparison in Mid-Latitude Regions. (**a**) Delay estimation in mid-latitude quiet channel; (**b**) Delay estimation in mid-latitude moderate channel; (**c**) Delay estimation in mid-latitude disturbed channel; (**d**) Absolute error in mid-latitude quiet channel; (**e**) Absolute error in mid-latitude moderate channel; (**f**) Absolute error in mid-latitude disturbed channel.

The test results for mid-latitude regions are summarized in Table 2 and Figure 3.

The simulation results in mid-latitude regions (Table 2) show that the MSIT algorithm controls the mean error within 0.011 ms across all states. In the MD scenario, the error of the Single-path DLL reaches 1.9899 ms, which is approximately 552 times larger than that of the MSIT (0.0036 ms). This massive discrepancy arises because the conventional DLL fails to correctly identify the first-arrival path during the acquisition stage. Standard delay-locked loops typically lock onto the correlation peak with the highest energy. In this disturbed scenario, the multipath interference causes the DLL to lock onto a stronger, delayed secondary path or the composite envelope rather than the true Line-of-Sight (LOS) path. Once falsely locked, the loop continues to track this delayed component, resulting in a deterministic bias equivalent to the inter-path delay.

3. Results in High-Latitude Regions

**Figure 4:**
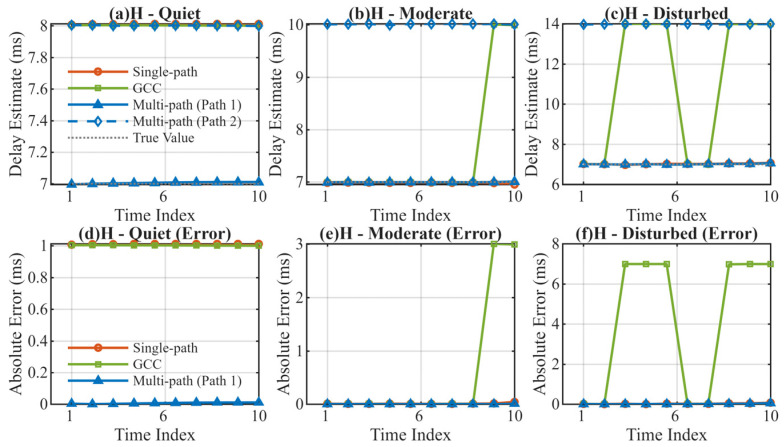
Delay Estimation Comparison in High-Latitude Regions. (**a**) Delay estimation in high-latitude quiet channel; (**b**) Delay estimation in high-latitude moderate channel; (**c**) Delay estimation in high-latitude disturbed channel; (**d**) Absolute error in high-latitude quiet channel; (**e**) Absolute error in high-latitude moderate channel; (**f**) Absolute error in high-latitude disturbed channel.

High-latitude regions are typically characterized by more severe ionospheric variations. The experimental results are shown in Table 3 and Figure 4.

Under the extreme environment of the high-latitude disturbed channel (HD), the GCC algorithm fails to track effectively, with an error reaching 4.2057 ms. Although the Single-path algorithm performs acceptably in the moderate state (HM), it exhibits large errors in the quiet state (HQ) due to failure in correctly locking onto the main path. Conversely, the MSIT algorithm demonstrates high robustness across all high-latitude scenarios, with a maximum mean error of only 0.0163 ms.

4. Comprehensive Analysis

A comprehensive analysis of the experimental data across the three latitudes indicates that the proposed MSIT algorithm consistently tracks the delay information of each path with high accuracy. In contrast, the Single-path algorithm frequently suffers from locking bias or misidentification of the main path due to inter-path interference. Similarly, the GCC algorithm exhibits significant jumps and deviations in estimation results due to the time-varying nature of shortwave channels and multipath superposition. By transforming multipath interference into resolvable observables, the MSIT algorithm significantly enhances delay estimation precision in complex ionospheric environments.

#### 4.1.2. Impact of Multipath Delay Interval on Estimation Performance

To further assess the algorithm’s capability in separating and tracking multipath signals, a simulation scenario was constructed with controlled multipath and noise injection. The dataset comprised 10 signal periods with a carrier frequency of 3.6 MHz, a symbol rate of 2.4 kHz, a sampling frequency of 10.8 MHz, and a signal period of 20 ms. The amplitude of the first path is set to 1 with a fixed delay of 7 ms, and the amplitude of the second path is 0.8. Both paths have a signal-to-noise ratio (SNR) of 20 dB. Comparative experiments were conducted with multipath delay differences set to 0.2 ms, 0.4 ms, 0.6 ms, 0.8 ms, 1 ms, and 2 ms, respectively.

**Figure 5:**
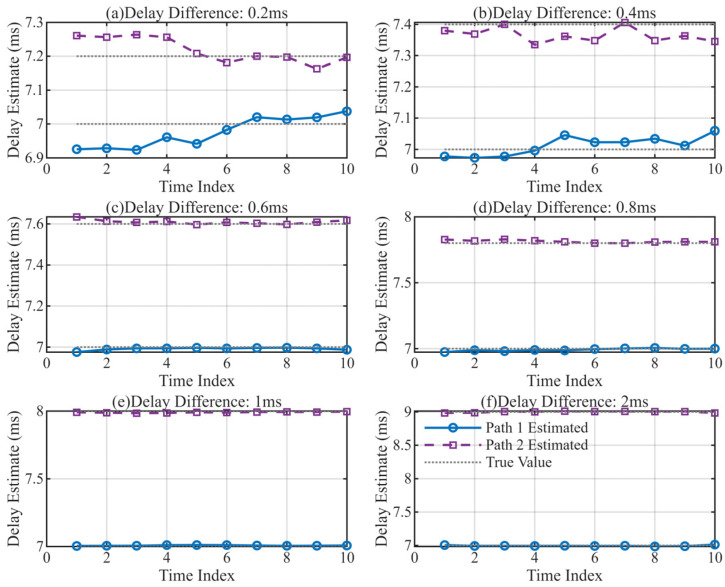
Multipath Delay Estimation Results. (**a**) Results at 0.2 ms delay difference; (**b**) Results at 0.4 ms delay difference; (**c**) Results at 0.6 ms delay difference; (**d**) Results at 0.8 ms delay difference; (**e**) Results at 1 ms delay difference; (**f**) Results at 2 ms delay difference;.

**Figure 6:**
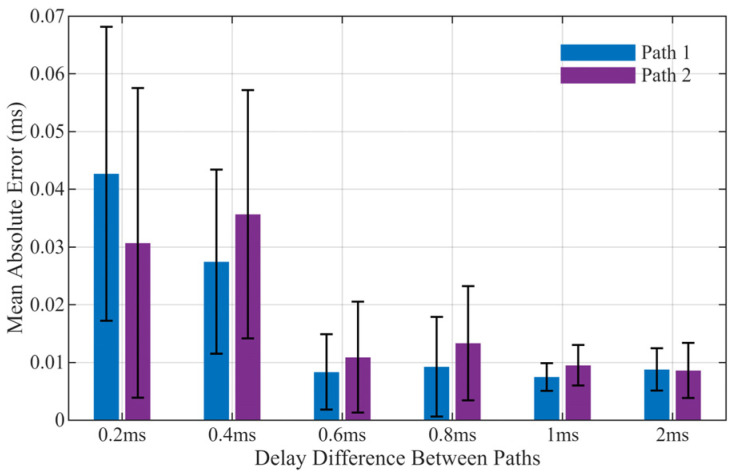
Mean Delay Errors Under Different Delay Intervals.

The multipath delay estimation results are shown in Figure 5, and the distribution of mean delay errors is shown in Figure 6. Experimental results indicate that with the adoption of the MUSIC super-resolution initialization method, the system achieves the ability to distinguish multipath components with delay intervals below 0.5 chip duration (0.2 ms and 0.4 ms), which was unattainable with traditional non-super-resolution initialization approaches.

Notably, for sub-0.5-chip delay interval scenarios where MUSIC initialization results deviate due to noise, the proposed Multipath Separation and Independent Tracking (MSIT) method demonstrates strong error correction and convergence capabilities. As the tracking process proceeds, the closed-loop tracking architecture (integrating MLE-based separation and independent DLL/PLL loops) gradually suppresses the noise-induced bias of the initial MUSIC estimates, and the delay values of each path will converge to the true delay with continuous iteration.

**Figure 7:**
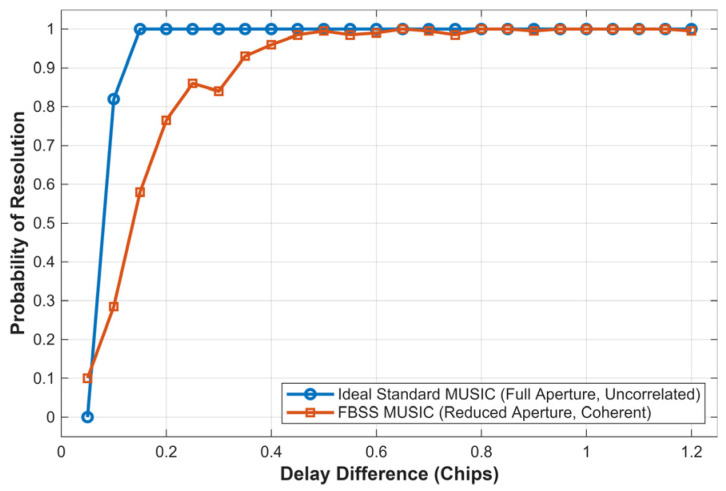
Probability of resolution versus multipath delay difference. The comparison between the ideal full-aperture MUSIC (blue) and the proposed FBSS-MUSIC (orange) quantifies the resolution degradation caused by the spatial smoothing technique.

It is important to note that the resolution capability of the initialization stage is physically constrained by the spatial smoothing technique required for coherent HF signals. As illustrated in Figure 7, we conducted a Monte Carlo analysis to quantify this impact. The ‘Ideal Standard MUSIC’ (blue curve) utilizes the full array aperture (M = 60) with uncorrelated sources, achieving a resolution limit of approximately 0.1 chips. However, in real-world HF channels where multipath components are fully coherent, Forward-Backward Spatial Smoothing (FBSS) is mandatory to restore the rank of the covariance matrix. This process reduces the effective aperture (L < M), resulting in a ‘Resolution Loss’, as shown by the orange curve.

Despite this degradation, the FBSS-MUSIC algorithm maintains a resolution probability of over 85% for delay differences greater than 0.25 chips. This initialization precision is sufficient for the subsequent MSIT tracking loops to lock onto the signal peaks. Once locked, the iterative MLE-based separation module (described in Section 3.2.1) further refines the estimates, effectively compensating for the initial resolution limitation.

#### 4.1.3. Algorithm Stability Verification

**Figure 8:**
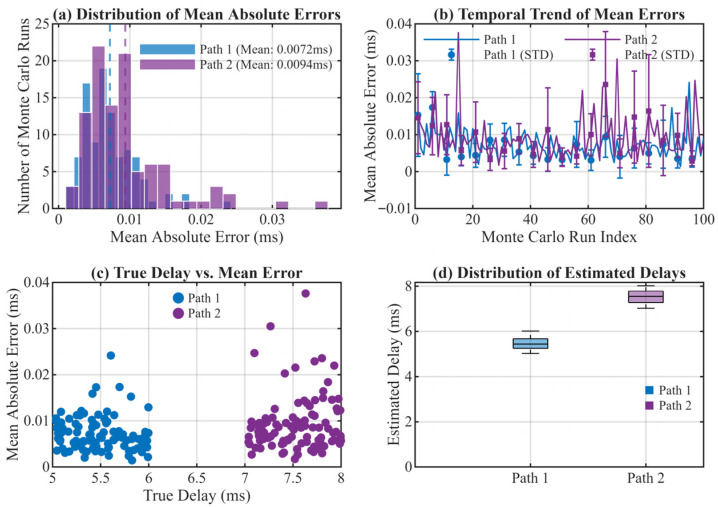
Stability Analysis Results. (**a**) Histogram of mean absolute errors; (**b**) Temporal trend of mean errors; (**c**) Scatter plot of true delay vs. mean error; (**d**) Boxplot of estimated delays.

The stability and robustness of the algorithm were validated through 100 Monte Carlo simulations. In each iteration, two paths were configured: the first with a random delay between 5–6 ms, and the second with a random delay between 7–8 ms, while the remaining settings were the same as those in Section 4.1.2. The mean absolute delay error of each experiment was used as the stability evaluation index. The results are as shown in Figure 8.

Figure 8a shows a histogram of the mean absolute error distribution. The errors of both paths are concentrated in the low-error region, and the distribution of the first path is more compact, indicating that the algorithm may achieve more stable estimation accuracy for paths with higher energy; Figure 8b is a temporal trend diagram of mean errors. The error sequences of both paths fluctuate slightly around low error values without obvious mutations or trend changes, indicating good consistency of the algorithm under different random scenarios; Figure 8c is a scatter plot of true delay vs. mean error. All data points are randomly distributed without obvious correlation, verifying that the algorithm’s performance is not affected by specific delay values; Figure 8d is a boxplot of delay estimates. The narrow box width and absence of outliers indicate small dispersion and significant central tendency of the estimated values, and the median is close to the midpoint of the true delay interval, proving the unbiasedness and stability of the algorithm.

In summary, the proposed MSIT architecture for shortwave signal time-of-arrival (TOA) estimation exhibits minimal estimation errors, high statistical consistency, and robust adaptability to random delay variations. These results collectively validate the algorithm’s reliability and highlight its significant potential for practical applications in high-precision sensor networks and communication systems where multipath propagation is a dominant source of error.

#### 4.1.4. Robustness Analysis Against Noise and Multipath Bias

To further evaluate the robustness of the proposed algorithm under varying noise levels, we conducted a sensitivity analysis of the estimation accuracy with respect to the Signal-to-Noise Ratio (SNR). The simulation was set up with a fixed multipath delay difference of 0.4 ms (approximately one chip width), which represents a challenging scenario close to the Rayleigh resolution limit. The SNR ranged from −10 dB to 20 dB, while the remaining settings were the same as those in Section 4.1.2.

**Figure 9:**
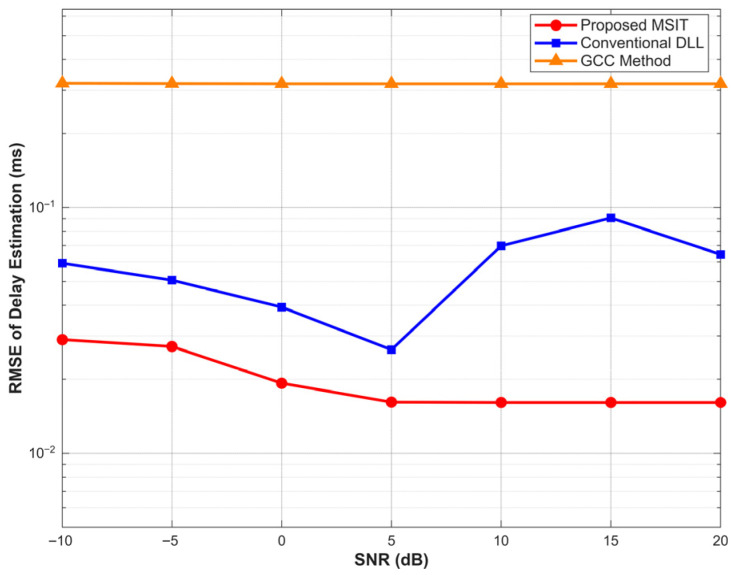
RMSE of delay estimation versus SNR under 0.4 ms multipath interval.

The Root Mean Square Error (RMSE) of the delay estimation for the three algorithms is illustrated in Figure 9.

The results reveal fundamental differences in the error mechanisms of the three methods. The GCC algorithm exhibits a consistently high estimation error that remains virtually constant across the entire SNR range. This flat trajectory confirms that the cross-correlation method is entirely constrained by the Rayleigh resolution limit rather than thermal noise; the algorithm fails to resolve the coherent multipath components, resulting in a persistent lock on a spurious correlation peak regardless of signal strength.

More notably, the Conventional DLL displays a non-monotonic error profile where the RMSE paradoxically increases in the high-SNR regime, specifically peaking between 10 dB and 15 dB. This phenomenon indicates that the standard tracking loop is bias-limited rather than noise-limited. In low-SNR conditions, random noise dithering provides a slight averaging effect; however, as the channel becomes cleaner, the loop locks more tightly onto the distorted composite envelope formed by the overlapping signals. Consequently, the systematic bias caused by the multipath superposition becomes the dominant error source, preventing any improvement in accuracy.

In sharp contrast, the proposed MSIT algorithm demonstrates superior robustness. The RMSE decreases monotonically as the SNR improves from −10 dB to 5 dB and subsequently stabilizes at a minimal error floor. This performance validates that the iterative cancellation mechanism effectively decouples the coherent multipath components, thereby transforming the interference into resolvable observables and allowing the system to fully capitalize on improved signal quality to minimize tracking errors.

### 4.2. Experimental Results

To validate the practical performance of the proposed algorithm, a field experiment was conducted involving shortwave signal transmission from Xi’an and reception in Ürümqi. The transmitter adopted 8PSK modulation, with 192 symbols as one transmission period, a carrier frequency of 12.36 MHz, and a signal bandwidth of 9.6 kHz. At the receiver side, a redundant data acquisition strategy was adopted to ensure reliability. Two dual-channel shortwave reception devices were deployed, effectively providing four potential receiving ports connected to collocated antennas. During the measurement campaign, valid signal streams were successfully acquired from three independent antenna channels simultaneously (one channel was excluded due to hardware instability). The sampling rate was set to 120 MHz, and 1 s of continuous data from these three synchronized channels was selected for comparative analysis. This multi-channel configuration allows for the verification of the algorithm’s stability across independent hardware paths under identical ionospheric conditions.

In the experiment, the performance of the proposed multipath delay estimation algorithm was compared with that of the traditional generalized cross-correlation (GCC) algorithm and the single-path delay estimation algorithm.

Since the actual propagation delay in field environments dynamically varies with real-time ionospheric scintillation and layer movement, its true value is unknown, so the practicality and reliability of the proposed algorithm were evaluated using three quantitative metrics: output stability, defined as the standard deviation of delay estimates over time; multipath identification capability, quantified by the number of valid paths correctly detected; and branch energy distribution consistency, assessed by the degree of match between measured I/Q and E/P/L energy ratios and the theoretical characteristics of the delay-locked loop (DLL).

The delay estimation results of the proposed MSIT algorithm were compared against the traditional Generalized Cross-Correlation (GCC) and the standard Single-Path DLL methods. The results for the three receiving channels are presented in Figure 9.

**Figure 10:**
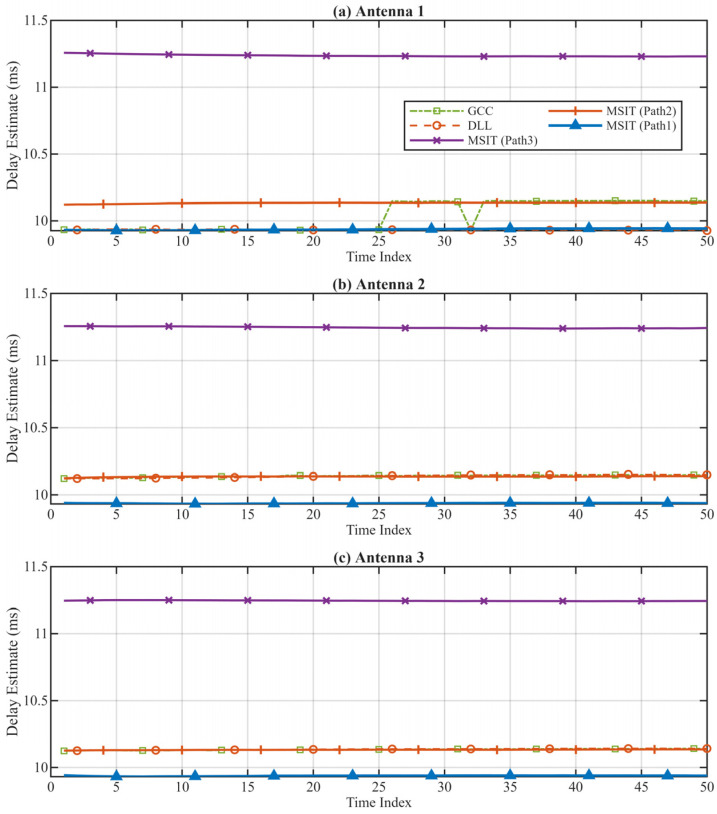
Comparison of Measured Delay Estimation Results. (**a**) Results of Antenna 1; (**b**) Results of Antenna 2; (**c**) Results of Antenna 3.

Figure 10 provides a direct performance comparison in terms of precision, stability, and multipath resolution—key metrics for sensor signal processing. The GCC algorithm exhibits poor stability (high jitter) and resolution (uncontrolled mode hopping), as it cannot decompose coherent components. The Single-Path algorithm achieves stability at the cost of critical resolution, outputting a single aggregate parameter that is blind to the multipath structure, which is inadequate for high-fidelity sensing.

In sharp contrast, the proposed MSIT algorithm successfully separates and stably tracks three distinct propagation paths (labeled Path 1, Path 2, Path 3). The ability to continuously resolve and track these closely spaced paths in a real-world scenario demonstrates the method’s core strength: transforming ambiguous multipath interference into stable, discrete observables. This provides a high-fidelity, multi-dimensional dataset that is essential for downstream applications requiring precise channel characterization, such as sensor network localization, channel diagnostics, or, indeed, ionospheric remote sensing.

**Figure 11:**
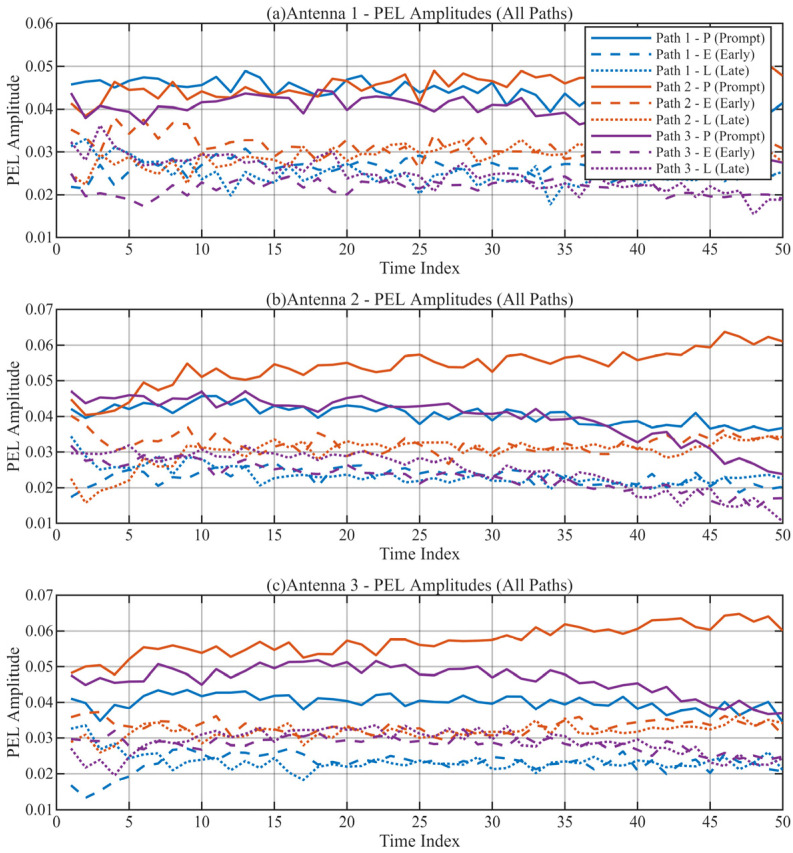
E, P, L Branch Integration Values. (**a**) Path PEL responses for Antenna 1; (**b**) Path PEL responses for Antenna 2; (**c**) Path PEL responses for Antenna 3.

**Figure 12:**
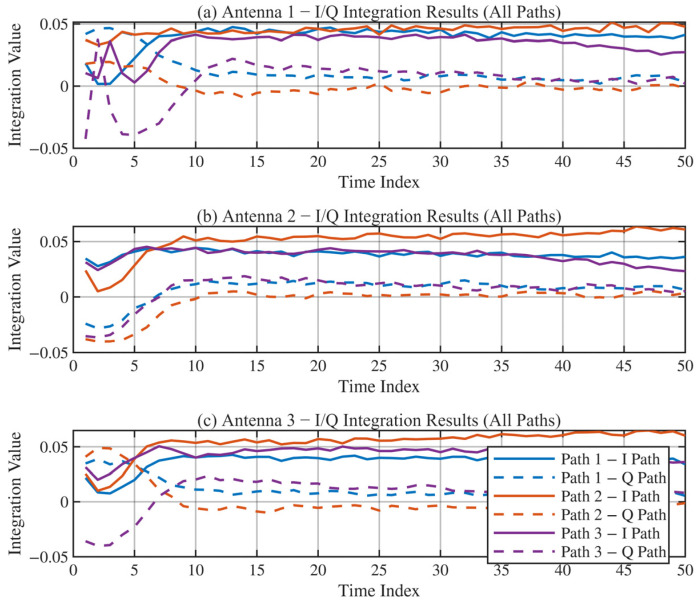
I/Q Branch Integration Values. (**a**) Antenna 1: I/Q Integration of All Paths; (**b**) Antenna 2: I/Q Integration of All Paths; (**c**) Antenna 3: I/Q Integration of All Paths.

To confirm the internal reliability of the tracking loops, we analyzed the correlation outputs. Figure 11 and Figure 12 display the integration values for the Early/Prompt/Late (E/P/L) code branches and the In-phase/Quadrature (I/Q) carrier branches, respectively.

In a perfectly locked loop, the Prompt branch energy should be maximum, and the Quadrature branch energy should be near zero. As shown in Figure 11, the Prompt (P) branch amplitude is approximately twice that of the Early (E) and Late (L) branches across all three paths, indicating precise code alignment. Furthermore, Figure 12 shows that the signal energy is almost entirely concentrated in the I-branch, while the Q-branch integral remains near zero. This confirms that the independent PLLs have successfully tracked the carrier phase of each path, effectively removing the Doppler rotation [26,27].

**Figure 13:**
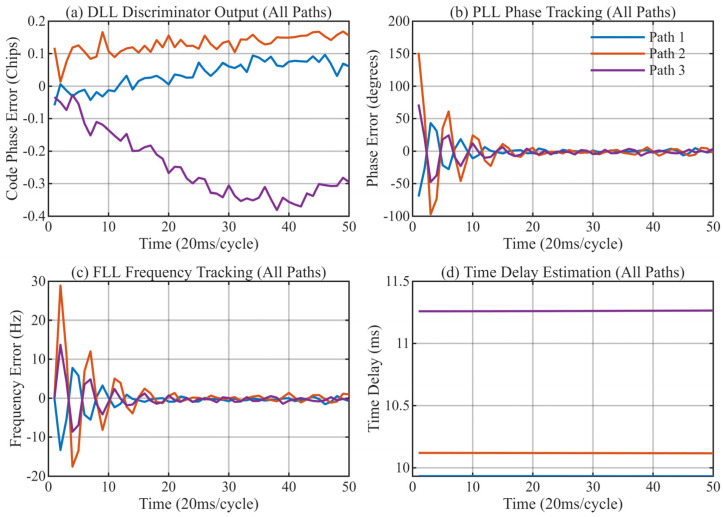
Antenna 1: Tracking performance of the MSIT algorithm. (**a**) DLL discriminator output for 3 paths; (**b**) PLL tracking results; (**c**) FLL tracking results; (**d**) Final delay estimation of 3 paths.

**Figure 14:**
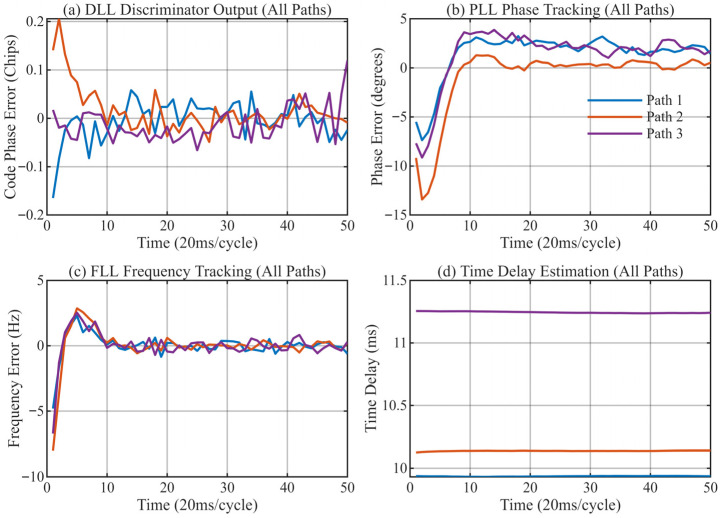
Antenna 2: Tracking performance of the MSIT algorithm. (**a**) DLL discriminator output for 3 paths; (**b**) PLL tracking results; (**c**) FLL tracking results; (**d**) Final delay estimation of 3 paths.

**Figure 15:**
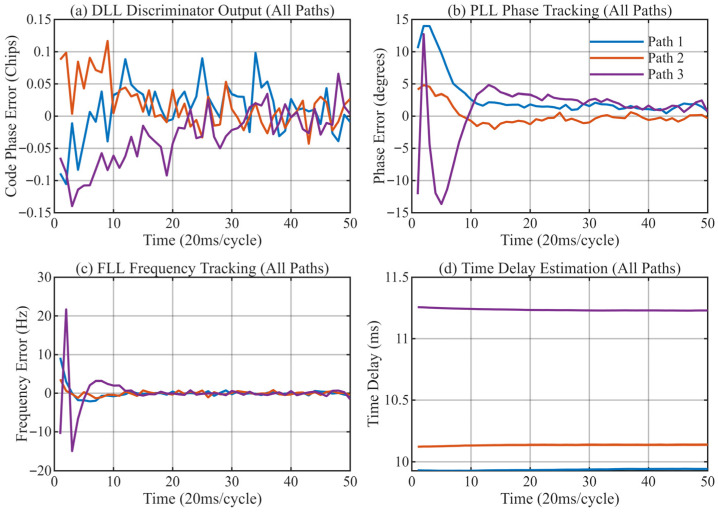
Antenna 3: Tracking performance of the MSIT algorithm. (**a**) DLL discriminator output for 3 paths; (**b**) PLL tracking results; (**c**) FLL tracking results; (**d**) Final delay estimation of 3 paths.

**Figure 16:**
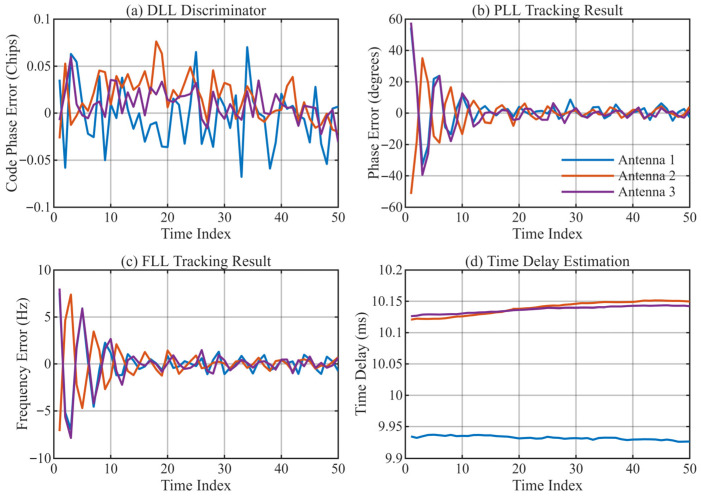
Tracking performance of the single-path algorithm. (**a**) DLL discriminator output for 3 antennas; (**b**) PLL tracking result of antennas; (**c**) FLL tracking result of antennas; (**d**) Final delay estimation of antennas.

Figure 13, Figure 14, Figure 15 and Figure 16 provide a detailed view of the transient convergence and steady-state tracking behavior. Both algorithms stabilize after approximately 20 signal cycles (approx. 0.4 s). We performed a statistical analysis on 30 consecutive cycles in the steady state (cycles 21–50), summarizing the Mean Delay and Standard Deviation (STD) in Table 4.

Table 4 presents the statistical results of the measured delay estimation. It is worth noting that due to the pre-filtering of the measured signal, the in-band SNR reaches 43 dB, and with the bandwidth increasing to 9.6 kHz, the calculated theoretical CRLB is extremely low (approximately $2.1\times 10^{-5}$ ms). However, the actual estimation Standard Deviation (STD) is around 0.0005—0.003 ms. This discrepancy indicates that in practical high-SNR HF channels, the dominant error sources are no longer Additive White Gaussian Noise (AWGN), but rather micro-multipath jitter caused by rapid ionospheric variations and residual synchronization errors in the transceiver system. Nevertheless, the stability of the MSIT algorithm remains significantly superior to traditional methods.

The statistical results demonstrate the enhanced precision of the proposed method: By separating mutual interference, the MSIT achieves an estimation STD between 0.0005–0.0032 ms. This represents a reduction of 47–84% compared to the Single-Path method (STD: 0.0019–0.0038 ms) and is markedly lower than that of the GCC method. Crucially, the MSIT stably resolves and tracks 3 propagation paths, whereas the Single-Path algorithm consistently locks onto only the dominant one. This capability to reliably recover the multi-path structure significantly improves data availability and signal integrity for high-precision sensing.

This comprehensive validation demonstrates that the MSIT architecture not only improves sensor measurement precision but, more importantly, transforms multipath interference into a resolvable, multi-dimensional dataset suitable for robust signal processing in complex monitoring environments.

### 4.3. Computational Complexity Analysis

To assess the feasibility of deploying the MSIT architecture on resource-constrained sensor nodes, we analyze the computational complexity in terms of Floating Point Operations (FLOPs). The processing flow is divided into two distinct phases: Initial Acquisition and Steady-State Tracking.

#### 4.3.1. Initial Acquisition Phase

The subspace-based MUSIC algorithm is employed only once at the initialization stage to estimate the number of paths and coarse delays. While the Eigenvalue Decomposition (EVD) of the covariance matrix (M×M) has a complexity of $O\left(M^{3}\right)$, this is a one-shot operation. It does not impose a continuous burden on the processor during real-time transmission.

#### 4.3.2. Steady-State Tracking Phase

During the continuous tracking phase, the computational load is dominated by the Iterative Least Squares (LS) estimation in the separation module (Equation (13)) and the parallel DLL/PLL discriminators. The complexity of the LS estimator scales with $O\left(N^{3}+N^{2}L\right)$, where N is the number of multipath components and L is the sample length per update period. Crucially, in HF ionospheric channels, the number of resolvable paths is typically small (typically N ≤4). Consequently, the dimension of the matrix inversion is minimal, making the real-time computational cost comparable to standard DLL architectures and significantly lower than GCC methods, which require frequent large-scale FFT operations ($O\left(L\log L\right)$) for peak searching.

Table 5 summarizes the complexity comparison, demonstrating that the proposed MSIT achieves high precision with a manageable computational overhead suitable for FPGA or DSP implementation.

## 5. Discussion

The results of this study demonstrate that the MSIT framework significantly enhances Time-of-Arrival (TOA) estimation accuracy in HF sensor networks by resolving multipath interference rather than suppressing it. In contrast to conventional Generalized Cross-Correlation (GCC) or single-path tracking algorithms—which treat multipath components as self-interference and suffer performance degradation when delay differences approach the Rayleigh resolution limit—the proposed iterative separation mechanism effectively isolates distinct signal components. This capability enables the system to accurately track weaker or overlapping paths while mitigating the bias introduced by envelope distortion, thereby reducing the mean delay error by approximately two orders of magnitude in simulations. These findings validate the hypothesis that high-precision acquisition in narrowband channels can be achieved by decoupling the channel impulse response into discrete, trackable elements, rather than relying on aggregate channel statistics.

From a practical sensing perspective, the proposed algorithm demonstrates superior robustness in non-stationary environments—a critical requirement for autonomous monitoring systems. Comparative analysis across low-, mid-, and high-latitude scenarios indicates that the closed-loop feedback architecture effectively prevents ‘mode hopping’ and lock loss, issues frequently observed in traditional methods during ionospheric disturbances. Field experiments conducted on the Xi’an–Ürümqi link further corroborate this stability; the system successfully tracked multiple propagation modes simultaneously, achieving a reduction in standard deviation of 47–84% compared to single-path methods. These findings suggest that the hybrid architecture—combining super-resolution initialization (MUSIC) with efficient parallel tracking—successfully balances computational cost with the need for real-time resolution, rendering it suitable for deployment in continuous monitoring sensors.

While the current model primarily assumes Additive White Gaussian Noise (AWGN), HF channels frequently experience impulsive noise (e.g., atmospheric lightning). Notably, the proposed MSIT architecture exhibits inherent robustness against moderate impulsive interference. This resilience is attributed to the integration effect of the correlators and the low-pass nature of the tracking loop filters, which effectively smooth out transient high-amplitude spikes that typically disrupt snapshot-based methods like GCC. For environments with extreme impulsive disturbances, future implementations could further enhance performance by replacing the LS estimator in the separation module with robust M-estimators.

Despite these advantages, the current interference cancellation relies on the assumption of linear superposition, which may limit performance under conditions of extreme non-linear ionospheric distortions or non-Gaussian impulsive noise. Future research will focus on integrating adaptive filtering techniques, such as deep learning-based Kalman filters, to dynamically adjust tracking loop bandwidths based on real-time signal quality. Additionally, future work will explore the implementation of the iterative separation module on FPGA platforms to evaluate the maximum number of trackable paths in resource-constrained hardware, further extending the applicability of this method in compact HF sensor nodes.

## Figures and Tables

**Table 1:** Mean delay estimation errors in low-latitude regions (ms).

Channel State	MSIT	DLL	GCC
Quiet (LQ)	0.0056	0.0101	0.0141
Moderate (LM)	0.0052	0.0066	0.0095
Disturbed (LD)	0.0030	0.0103	1.2030
CRLB	0.0023	0.0023	0.0023

**Table 2:** Mean delay estimation errors in mid-latitude regions (ms).

Channel State	MSIT	DLL	GCC
Quiet (MQ)	0.0032	0.4959	0.4971
Moderate (MM)	0.0108	1.0019	0.9961
Disturbed (MD)	0.0036	1.9899	1.1990
CRLB	0.0023	0.0023	0.0023

**Table 3:** Mean delay estimation errors in high-latitude regions (ms).

Channel State	MSIT	DLL	GCC
Quiet (HQ)	0.0072	1.0113	1.0027
Moderate (HM)	0.0035	0.0124	0.6030
Disturbed (HD)	0.0163	0.0285	4.2057
CRLB	0.0023	0.0023	0.0023

**Table 4:** Time delay estimation analysis results.

Algorithm	Antenna 1		Antenna 2		Antenna 3	
	Mean (ms)	STD (ms)	Mean (ms)	STD (ms)	Mean (ms)	STD (ms)
MSIT (path1)	9.9398	0.0032	9.9377	0.0013	9.9381	0.0005
MSIT (path2)	10.1357	0.0005	10.1373	0.0011	10.1348	0.0011
MSIT (path3)	11.2307	0.0013	11.2410	0.0024	11.2430	0.0011
DLL	9.9307	0.0020	10.1471	0.0038	10.1410	0.0019
GCC	10.1043	0.0881	10.1453	0.0021	10.1387	0.0027

**Table 5:** Computational complexity comparison.

Algorithm	Initialization Cost	Tracking Cost (Per Iteration)	Scalability
GCC	Low (FFT)	Medium (FFT + Peak Search)	$O\left(L\log L\right)$
DLL	Low (FFT)	Low (Correlators)	$O\left(L\right)$
MSIT	High (Covariance + EVD)	Medium (Correlators + N×N Matrix Inv.)	$O\left(L\cdot N+N^{3}\right)$

## Data Availability

The supporting data for this study can be obtained upon request from the corresponding author. Due to privacy concerns involving the participants, these data are not publicly available.
